# Distribution of Mercury and Methylmercury in Farmland Soils Affected by Manganese Mining and Smelting Activities

**DOI:** 10.3390/ijerph191610288

**Published:** 2022-08-18

**Authors:** Yongjiang Zhang, Xian Zhou, Weibin Ma, Deliang Yin, Yongmin Wang, Cheng Zhang, Dingyong Wang

**Affiliations:** 1 Department of Environment and Quality Test, Chongqing Chemical Industry Vocational College, Chongqing 401220, China; 2 College of Resources and Environment, Southwest University, Chongqing 400715, China; 3 Key Laboratory of Karst Georesources and Environment, Ministry of Education, Guizhou University, Guiyang 550025, China

**Keywords:** manganese-related activities, soil, mercury, methylmercury, influencing factors, environmental hazard

## Abstract

Manganese (Mn)-related activities would affect the mercury (Hg) cycling in farmlands, whereas this was not well understood. Here, one of the largest Mn ores in China was selected to study the effects of Mn-related activities on the accumulation and distribution of total Hg (THg) and methylmercury (MeHg) in farmland soils. The soil THg concentrations in the mining area were 0.56 ± 0.45, 0.56 ± 0.45, 0.53 ± 0.44, and 0.50 ± 0.46 mg kg^−1^ in the 0–10, 10–20, 20–30, and 30–40 cm layers, respectively, while they were increased to 0.75 ± 0.75, 0.72 ± 0.60, 0.62 ± 0.46, and 0.52 ± 0.38 mg kg^−1^ in the smelting area. Similarly, the soil MeHg concentrations in the smelting area were also elevated by 1.04–1.34 times as compared to those in the mining area. Concentrations of THg (0.59 ± 0.50 mg kg^−1^) and MeHg (0.64 ± 0.82 μg kg^−1^) in soils were higher than the regional background value but lower than in vicinal Hg-mining areas, while they were largely elevated at the intersection of two rivers in the smelting area. Significant positive Mn-THg relationship (*p* < 0.01) and negative Mn-MeHg relationship (*p* < 0.01) favored the conclusion that soil Mn could promote Hg accumulation while inhibiting MeHg production. Approximately 70% of soil Hg was distributed in the residual phase, and the environmental hazard was not elevated according to a geochemical model. Overall, mining and smelting activities of Mn ores have resulted in obvious and distinct effects on the accumulation and methylation of Hg in farmland soils, but the environmental hazards are currently manageable.

## 1. Introduction

Mercury (Hg) is a hazardous heavy metal that can be methylated to highly neurotoxic and bioaccumulative methylmercury (MeHg) in aquatic and terrestrial environments [1,2]. Recent studies in Hg mining areas have revealed that farmland soils in these areas suffer from higher Hg contamination and MeHg production than other areas and that the crops and vegetables grown in these areas accumulate extremely high concentrations of MeHg [3,4]. This contamination is a serious risk for local residents [3]. Consequently, Hg cycling in Hg-contaminated farmlands is of concern [5].

In addition to natural processes, anthropogenic sources such as mining and smelting of metal ores, sewage irrigation, and agricultural activities can introduce Hg to farmland soil [6]. Ore mining and smelting activities are the key contributors in regional farmland areas [6]. The Hg concentrations in agricultural soils around Hg mining areas could reach 187.8 mg kg^−1^ [7]. After Hg-containing materials enter the soil, they are subjected to many biogeochemical reactions, including Hg methylation, which results in high MeHg accumulation in the edible parts of crops [8]. Elevated MeHg levels in the food chain and agricultural products are frequently observed in Hg mining and smelting areas and pose high health risks to humans [6,9,10]. Recently, attention has been focused on Hg cycling in farmland soil located in Hg and gold mining areas. However, few studies have investigated the effects of mining and smelting of Mn ores. One study on Mn ore showed that it had a high content of Hg, which could be released to the environment via wastewater during smelting and have high bioavailability [11].

Xiushan country, one of the largest Mn mining and smelting areas in the world, is located in the southeast of Chongqing, China. Mining and electrolysis processes in this region have released large amounts of heavy metals into the soil via atmospheric deposition, irrigation, and slag leaching [12]. Some studies have investigated the environmental impacts of mining and smelting of Mn ore in this region [13,14], and high Hg concentrations have been detected in Mn ore and electrolytic Mn residues [13]. Consequently, there are elevated total Hg (THg) and MeHg concentrations in the water and sediments near this mining and smelting area [13]. Little information is available about the regional distributions of THg and MeHg in farmland soils affected by Mn mining and smelting activities. Two studies conducted in the Xiushan Hg mining area, downstream from the Mn mining area, have shown that paddy soils in this area are polluted with elevated Hg concentrations [15,16]. However, these studies did not investigate the contributions of Mn mining and smelting activities, which are dominant pollution sources. We hypothesized that mining and smelting activities would (1) increase the total Hg and MeHg accumulation in farmlands, (2) differentiate the distribution of total Hg and MeHg, and (3) increase potential environmental hazards. Further research is required to study the pollution levels, distribution characteristics, and environmental hazards of Hg in farmland soils affected by Mn mining and smelting.

In order to verify the above-mentioned hypothesis, in this study, field investigations were performed to collect soil profile samples from different farmland types, including paddies, drylands, and paddy-upland rotations in the Mn mining and smelting areas. Total Hg, MeHg, and geochemical fractionation of Hg in soils were measured, and soil organic matter (SOM), pH, cation exchange capacity (CEC), and elements were synchronously measured to find the influencing factors regulating the distribution of soil THg and MeHg. Thereafter, we expected to achieve several objectives, including: (1) to understand the spatial distributions and accumulation characteristics of THg and MeHg in soils; (2) to find the influencing factors regulating the distribution and accumulation of THg and MeHg in soils; and (3) to identify the geochemical fractionation and the potential environmental hazard of Hg in soils. This research would improve our understanding of the environmental impacts of large-scale mining and smelting of Mn ore.

## 2. Materials and Methods

### 2.1. Studying Area

**Figure 1:**
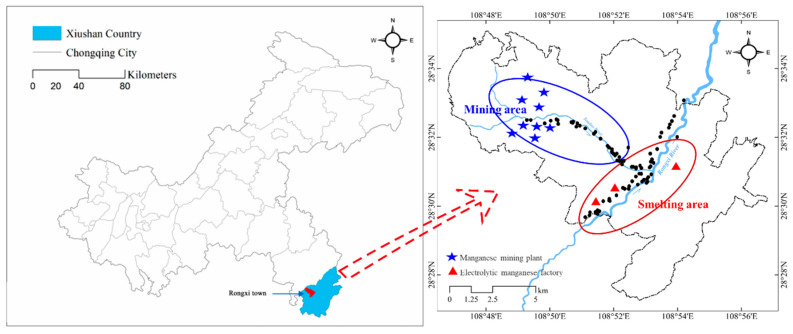
Sampling sites in the Mn mining and smelting areas.

Xiushan county (28°9′43″–28°53′5″ N, 108°43′6″–109°18′58″ E) is located in Chongqing, Southwestern China (Figure 1). The annual average temperature is 16 °C, and the annual precipitation is approximately 1325 mm. This area has a subtropical humid monsoon climate. The highest elevation is 1641.3 m above sea level. The study area is rich in Mn ore and is located within the “Manganese Triangle”, where there is the world’s largest Mn mining and electrolytic manufacturing base [17]. Soil and crops in this area suffer from Mn pollution [17]. Two independent functional zones before the mining and smelting (electrolytic techniques) areas were selected around the upstream of Toudao River and Rongxi River (Figure 1). Farmland land is widely distributed in this region, and environmental problems caused by mining and smelting should be evaluated.

### 2.2. Sampling and Preparation

In May 2017, samples were collected from paddies (*n* = 24), drylands (*n* = 58), and paddy-upland rotations (*n* = 5) in the mining and smelting areas (Figure 1). The farmland areas were distributed along the Toudao River, which was affected by mining, and the Rongxi River, which was affected by smelting. The geographical coordinates of the sampling sites were recorded using a hand-held GPS. At every site, soil profile samples were collected at depths of 0–10, 10–20, 20–30, and 30–40 cm by a steel profile sampler. A 500 g soil sample was collected from each soil layer and packed in a self-sealing polyethylene bag. The THg and MeHg concentrations, geochemical fractionation of Hg, and soil properties for these samples were measured. Parallel soil samples were collected in the field following a proportion of 10%. In total, 780 soil samples were collected. The collected soil samples were transported at 4 °C in a car refrigerator and then stored at −25 °C in the laboratory. Before analysis, the soil samples were freeze-dried at −60 °C, ground to powders with an agate mortar, passed through a 200-mesh nylon sieve, and then stored at −25 °C.

### 2.3. Chemical Analysis

The total Hg concentrations in the soil samples were directly determined using an automatic Hg analyzer (Hydra II C, Leeman Labs, Hudson, NH, USA) with a detection limit of 0.005 ng following Environmental Protection Agency Method 7473 (USEPA, 2007). MeHg concentrations in the soil samples were measured following a procedure that involved extraction with 20% CuSO_4_/HNO_3_ and CH_2_Cl_2_, water back-extraction with heating, and derivatization with sodium tetraethylborate. The generated diethyl mercury and methyl ethyl mercury were captured by a Tenax trap, separated by isothermal gas chromatography, and then detected by cold vapor atomic fluorescence spectrometry according to Environmental Protection Agency Method 1630 (US EPA, 1998).

The soil pH value was measured using the potentiometric method [18]. The SOM content was determined by K_2_CrO_7_ oxidation titration combined with a volumetric technique [19]. The CEC was measured by exchanging cations in soil with hexamminecobalt trichloride and then analyzed by spectrophotometry [20].

An optimized Tessier’s sequential chemical extraction method was used to extract the Hg fractionation in the soil [13,21]. The extraction procedures are shown in Table 1. The concentrations of dissolved and exchangeable Hg (Hg-EX) and carbonate-bound Hg (Hg-CAR) were determined by cold vapor atomic fluorescence spectrometry [22]. The concentrations of Fe/Mn oxide-bound Hg (Hg-OX) and organic matter-bound Hg (Hg-OM) were determined by cold atomic absorption spectrometry [23]. The concentrations of residual Hg (Hg-RES) were determined by atomic fluorescence spectrometry [24].

Soil selenium (Se) was detected following a method described by Hailan et al. Briefly, 0.5 g of soil was weighed into a 50 mL glass colorimetric tube, and 10 mL of aqua regia was added. Next, the mixture was boiled in a water bath for 2 h and then replenished with ultrapure water before analysis by hydride generation atomic fluorescence spectrometry [23]. Soil Mn was extracted by HCl–HNO_3_–HClO_4_–HF wet digestion. The digestion solution was transferred to a 50mL volumetric flask and then analyzed by ICP-AES [25]. Samples for analysis of soil Fe and S were prepared by powder tableting, and the contents of Fe and S were determined by X-ray fluorescence spectrometry [26].

### 2.4. Quality Assurance and Quality Control

Quality assurance/quality control for the chemical analysis was conducted by analysis of duplicates and the standard reference materials GBW07405/GSS-5 (THg, 0.29 ± 0.03 mg kg^−1^, China) and GBW08308 (MeHg, 5.20 ± 0.70 ng g^−1^, China). The recovery ranges for THg and MeHg were 81%–116% and 84%–108%, respectively, and the relative standard deviations for all duplicate samples were <15%.

Statistical analysis of the data was performed using SPSS 19.0, Excel 2021, and Origin 2021. We first used the Kolmogorov–Smirnov test to find whether the overall data obeyed a certain normal distribution. Thereafter, the Kruskal–Wallis test was used to test the significant differences between data from different processing groups.

## 3. Results and Discussions

### 3.1. Soil Properties

Some of the soil properties are shown in Table 2. The soil pH values ranged from 4.16 to 7.93, and the proportion of pH values lower than 7 was 87.36%. These results indicated that the farmland soils in the Mn mining and smelting area were weakly acidic.

The SOM contents ranged from 2.70 to 64.85 g kg^−1^ and showed huge spatial variation. The average SOM contents in the 0–10, 10–20, 20–30, and 30–40 cm soil layers were 28.84 ± 9.93, 26.26 ± 8.50, 21.56 ± 8.69, and 16.93 ± 9.15 g kg^−1^, respectively. These results revealed that SOM accumulated in the topsoil, and there was an obvious soil profile distribution.

The soil CEC ranged from 3.37 to 17.35 cmol^+^ kg^−1^ in the Mn mining area. The average values in the 0–10, 10–20, 20–30, and 30–40 layers were 10.50, 10.04, 9.76, and 9.59 cmol^+^ kg^−1^, respectively. In the smelting area, the soil CEC ranged from 6.32 to 29.89 cmol^+^ kg^−1^, and the average values in the 0–10, 10–20, 20–30, and 30–40 cm soil layers were 12.13, 11.54, 11.08, and 10.89 cmol^+^ kg^−1^, respectively. These results indicate that surface soil has a higher CEC than other soil layers and that farmland soils in the smelting area have higher CECs than soil in the mining area.

The Mn, Fe, S, and Se concentration ranges were 85.16–10,858.12, 6088.50–71,869.28, 14.04–933.24, and 0.17–3.02 mg kg^−1^, respectively. The results showed a similar pattern to the SOM contents.

### 3.2. Distribution of THg in Farmland Soils

The THg concentrations in the farmland soils were 0.54 ± 0.45 and 0.63 ± 0.55 mg kg^−1^ in the mining and smelting areas, respectively. These values were higher to the background value of 0.069 mg kg^−1^ in agricultural soils in Chongqing [14] and also had an over-limit ratio of 20% compared to the risk screening value of the Chinese control standard [27]. Compared with other Hg mining areas in China, the concentrations in the present study are much lower than those in Xiushan (9.8 ± 17.5 mg kg^−1^) [15] and Wanshan (31.00 mg kg^−1^) [7] (Table S1). These results indicate that mining and smelting of Mn ore have resulted in obvious Hg accumulation in farmland soil in the study area. The Hg concentrations in soil in this area are related to irrigation of the land using Hg-contaminated river water [13].

**Figure 2:**
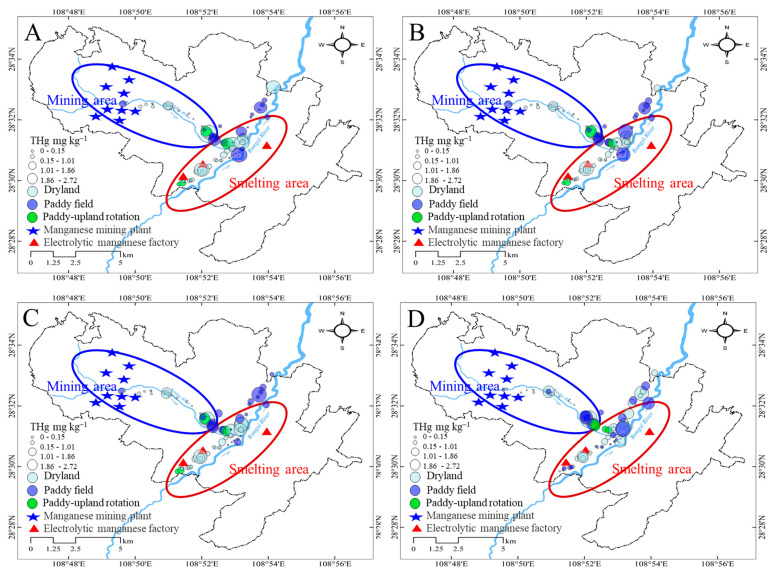
Spatial distribution of total Hg in different soil profiles in the farmlands at mining and smelting areas (**A**): 0–10 cm, (**B**): 10–20 cm, (**C**): 20–30 cm, and (**D**): 30–40 cm.

Regardless of the soil profiles, farmland soil located downstream of mining sites was subjected to less Hg accumulation. The THg concentrations were greatly elevated at the intersection of two rivers in the smelting area (Figure 2). The spatial distribution of Hg revealed that smelting activity could induce Hg accumulation in farmland soils.

**Figure 3:**
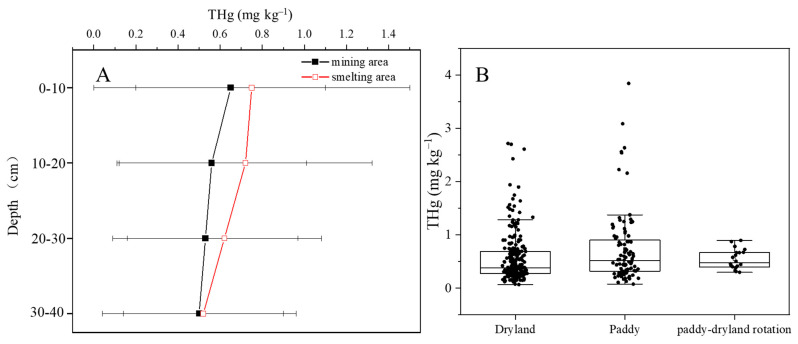
Distribution of total Hg in soil in different profiles (**A**) and land-used styles (**B**).

In both the mining and smelting areas, high spatial fluctuations in the soil layers were observed. This may be related to the high spatial heterogeneity of Hg concentrations in the irrigation water [13]. In the mining area, farmland soils had average THg concentrations of 0.56 ± 0.45, 0.56 ± 0.45, 0.53 ± 0.44, and 0.50 ± 0.46 mg kg^−1^ in the 0–10, 10–20, 20–30 cm, and 30–40 cm layers, respectively, and the differences between the soil profiles were not statistically significant (*p* > 0.05). In contrast, the average THg concentrations in the soil in the smelting area were high regardless of the soil profile and gradually reduced with increasing depth of the soil layer. This indicated that smelting activity had a larger contribution to Hg accumulation than mining activity, especially in the topsoil layer (Figure 3A).

The THg concentrations in the dryland, paddy, and paddy-upland rotation soils were 0.55 ± 0.46, 0.73 ± 0.67, and 0.54 ± 0.19 mg kg^−1^, respectively. Although the THg concentrations showed large spatial heterogeneity regardless of the land use, we found that paddy soils had the highest average Hg concentrations. This could be related to the higher irrigation frequency of paddies than other types of land, which would increase the input of Hg-containing materials (Figure 3B).

### 3.3. Distribution of MeHg in Farmland Soils

Soil MeHg concentrations in the mining area and smelting area were 0.36 ± 0.39 and 0.88 ± 1.00 μg kg^−1^, respectively. These concentrations were higher than background concentrations in the Chongqing area (0.41 ± 0.19 μg kg^−1^) [28], and lower than those in Hg mining areas in Xiushan (1.36 μg kg^−1^) [15] and Wanshan 2.8 μg kg^−1^ [7]. The ratio of MeHg to THg (%MeHg) in soils is an indicator of the Hg methylation efficiency [29]. In the mining area, this ratio was 0.08 ± 0.08%, which was significantly lower (*p* < 0.01) than that in the smelting area (0.26 ± 0.04%). This indicates that farmland soils in the smelting area have a higher capacity for Hg methylation and/or a higher input of exogenous MeHg than soils in the mining area. Similar results were obtained in a Hg smelting area in Wanshan, where high MeHg levels were attributed to the bioactivity of newly deposited atmospheric Hg [10]. In this Mn smelting area, the release of mineral-bound Hg in the electrolytic process may increase microbial in situ MeHg production and/or exogenous MeHg input via water irrigation.

**Figure 4:**
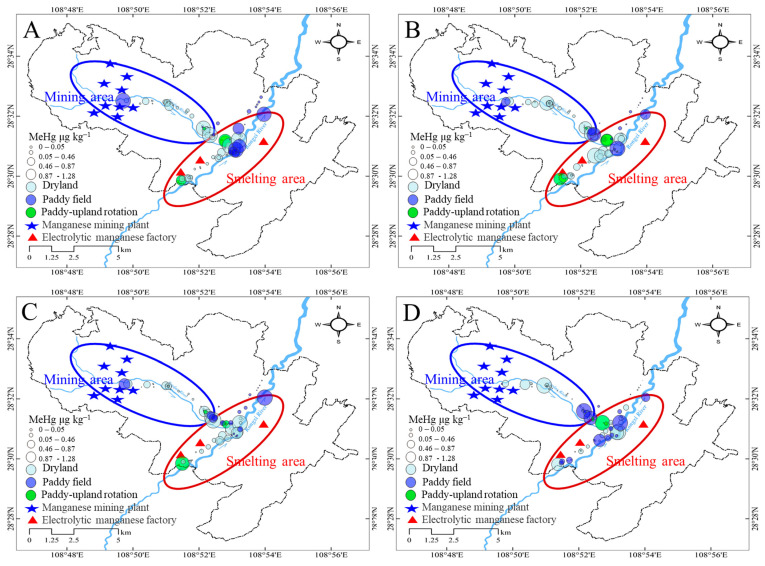
Spatial distribution of MeHg in different soil profiles in the farmlands at mining and smelting areas (**A**): 0–10 cm, (**B**): 10–20 cm, (**C**): 20–30 cm, and (**D**): 30–40 cm).

Regardless of the soil profiles, the MeHg concentrations in farmland soils located downstream of the mining and smelting sites decreased, which could be caused by purification processes in the stream that could reduce the input of methylated Hg [28]. Similar to the distribution of THg, the MeHg concentrations in soils were largely elevated at the intersection of two rivers adjacent to the smelting sites (Figure 4). This indicated that smelting activity could contribute to MeHg accumulation in the farmland soils.

**Figure 5:**
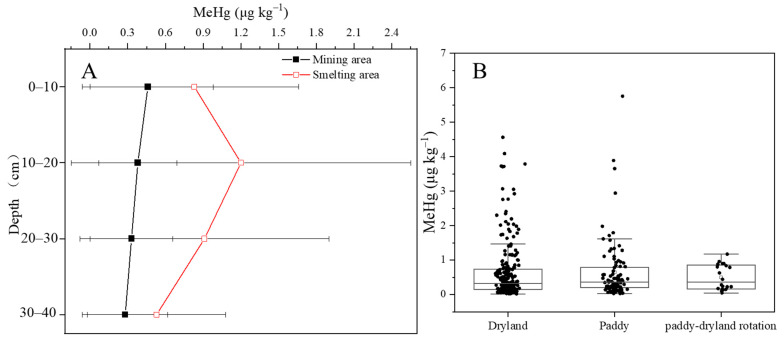
Distribution of MeHg in soil in different profiles (**A**) and land-used styles (**B**).

Similar to the THg distribution, high spatial heterogeneity was observed in the soil layers (Figure 3A and Figure 5A). In the mining area, the average soil MeHg concentrations were 0.46 ± 0.52, 0.38 ± 0.31, 0.33 ± 0.33, and 0.28 ± 0.34 μg kg^−1^ in the 0–10, 10–20, 20–30, and 30–40 cm soil layers, respectively, which showed a slight reduction in concentration with increases in soil depth. By contrast, in the smelting area, the concentrations increased to 0.83 ± 0.83, 1.22 ± 1.35, 0.93 ± 0.99, and 0.54 ± 0.55 μg kg^−1^ in the 0–10, 10–20, 20–30, and 30–40 cm soil layers (Figure 5A). A higher MeHg concentration was found in the soil sublayer, which was consistent with the SOM distribution.

In the mining area, the MeHg concentrations in the dryland, paddy, and paddy-upland rotation soils were 0.36 ± 0.31, 0.73 ± 1.09, and 0.71 ± 0.42 μg kg^−1^, respectively (Figure 5B). By contrast, in the smelting area, the MeHg concentrations in the dryland and paddy soils were 0.95 ± 0.95 and 0.65 ± 0.59 μg kg^−1^, respectively (Figure 5B). Paddy fields had the highest concentrations in the mining area, but drylands had the highest concentrations in the smelting area. These results indicate that different industrial activities have resulted in a complex accumulation of MeHg in farmland soil. Higher Hg methylation occurs in flooded paddies than in dryland areas [30], and we speculate that the MeHg input from water irrigation is an important source of MeHg in the soil.

### 3.4. Effects of Soil Properties on Hg Distribution

**Figure 6:**
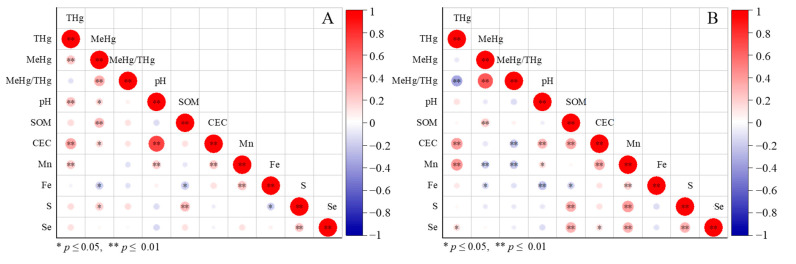
Correlation between Hg and soil physical and chemical properties in mining (**A**) and smelting areas (**B**).

In the mining area, total Hg showed significant relationships with pH, Mn, and CEC but no relationship with SOM (Figure 6A). By contrast, in the smelting area, total Hg had significant relationships with Mn and CEC but no relationship with pH and SOM (Figure 6B). Both Hg and Mn in farmland soils may have similar sources, such as Mn/Hg-containing wastewater [13], which would enhance their relationship in the smelting area. Our results indicate that smelting sources have greater contributions to Mn/Hg than mining sources. Although SOM has a positive role in the accumulation of Hg in soil [31], we could not identify a close relationship in this study. Moreover, because the CEC value reflects the soil cation adsorption capacity [32], we speculated that a high CEC would result in the retention of exogenous Hg in soil.

In the mining area, soil MeHg had a positive relationship with the THg concentration, pH, SOM content, CEC, and S concentration but had a negative relationship with the Fe concentration (Figure 6A). However, in the smelting area, soil MeHg only had a positive relationship with the SOM content, and its relationships with the Fe and Mn concentrations were negative (*p* < 0.05). It had no obvious relationship with the THg concentration (Figure 6B). Although SOM only has a minor role in the accumulation of Hg in soil, it can favor MeHg production by fueling Hg-methylating bacteria [33]. The lack of a relationship between the THg concentration and MeHg in the smelting area was interesting because this area may receive Hg from the electrolytic Mn plant (Figure 6B). The negative relationship between Mn and MeHg or %MeHg in the smelting area indicated that Mn released from the electrolytic Mn plant might inhibit Hg methylation in soil. Soil Fe has similar impacts on MeHg production with Mn. This may be because Fe/Mn oxides act as important binding ligands competing for soil Hg [6].

### 3.5. Potential Hazard Based on Geochemical Fractionation of Hg in Soil

**Figure 7:**
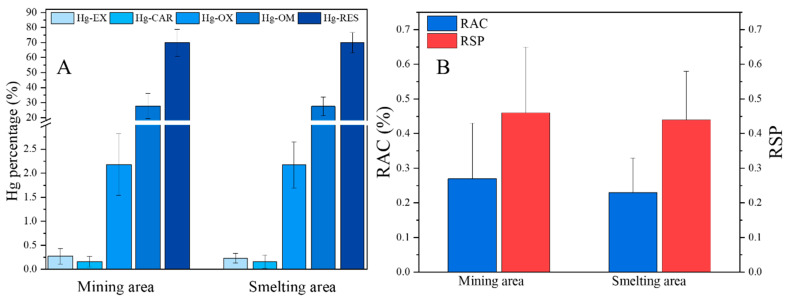
Geochemical fractionation of Hg in soil in the mining and smelting areas (**A**) and potential hazard of soil Hg based on RAC and RSP models (**B**).

To evaluate the hazard of Hg in the topsoil to humans or wildlife, we focused on the geochemical fractionation of Hg in soils at 0–10 cm. The contributions of Hg-EX, Hg-CAR, Hg-OX, Hg-OM, and Hg-RES were 0.27 ± 0.16%, 0.16 ± 0.11%, 2.18 ± 0.64%, 27.60 ± 8.38%, and 69.79 ± 8.97%, respectively, in the mining area. In the smelting area, the corresponding values were 0.23 ± 0.10%, 0.16 ± 0.14%, 2.17 ± 0.48%, 27.48 ± 6.32%, and 69.95 ± 6.75%. No obvious differences were observed between mining and smelting areas. Cluster analysis was conducted, and no difference between mining and smelting areas was found (Figure S1). The Hg predominantly accumulated in the residual phase, and the contribution of the bioavailable fraction was low (Figure 7A), although farmland soils in the smelting areas were found to have higher THg and MeHg levels.

Because geochemical models of Hg can provide further information about their bioavailability, potential hazards in this study were evaluated using the ratio of the secondary phase to the primary phase (RSP) and the risk assessment code (RAC). A hazard assessment was conducted using the geochemical fractionation of Hg (Figure 7B). Compared with the classification standards, the RAC values revealed soil Hg in the mining (0.27 ± 0.16%) and smelting (0.23 ± 0.10%) areas was not hazardous. Meanwhile, RSP values further confirmed this result, indicating that the environmental risk of Hg in the mining areas is less distinguished from that in the smelting areas. This low environmental hazard may be partially related to the high Mn input, which acts as an important inhibitor of Hg mobility (Figure 7).

## 4. Conclusions

The impacts and potential hazards of Mn mining and smelting on the accumulation and distribution of Hg in farmland soils were evaluated. Regardless of the soil profile, THg and MeHg concentrations in the farmland soils were elevated, especially in the smelting area. The concentrations of THg and MeHg in the soil in the smelting area were 0.63 ± 0.55 and 0.88 ± 1.00 μg kg^−1^, respectively. Regardless of soil profiles, the THg concentrations in the farmland soils in the smelting areas (0.63 ± 0.55 mg kg^−1^) were higher than those in the mining areas (0.54 ± 0.45 mg kg^−1^), while soil MeHg concentrations also increased from 0.36 ± 0.39 μg kg^−1^ in the mining area to 0.88 ± 1.00 μg kg^−1^ in the smelting area. There was obvious spatial heterogeneity with the THg and MeHg concentrations in the farmland soils, decreasing downstream of the mining sites but increasing at the intersection of two rivers in the smelting area. Soil properties had important and distinct effects on Hg behavior in the mining and smelting areas, among which Mn and Hg in soils may undergo synchronous accumulation, and high Mn concentrations tend to favor Hg accumulation but inhibit Hg methylation. Residual phase-bound Hg was the dominant geochemical fractionation of soil Hg (69.79 ± 8.97%), much larger than other Hg specials. Currently, geochemical models, including the ratio of the secondary phase to the primary phase and the hazard assessment code, show that the environmental hazards induced by mining and smelting activities are controllable.

## Figures and Tables

**Table 1:** Procedure of an optimized Tessier’s sequential chemical extraction method.

Procedure of Modified Tessier Method	Reagent	The Specific Methods
The water soluble and exchangeable (Hg-EX)	1mol/L Mg(NO_3_)_2_ (HNO_3_ regulates pH = 7.0)	Accurately weighing 2.0 g soil samples, adding16 mL extractant, shaking at room temperature for1h, centrifugal 20 min (3500 r/min), supernatant through 0.45 μm microporous membrane, analysis. The residue was washed twice with 8ml ultrapure water.
The bound to carbonate (Hg-CAR)	1mol/L NaAc (CH_3_COOH regulates pH = 5.0)	After the first step, 16 mL extract was added to the residue, shaking at room temperature for 5 h, centrifuged for 20 min, the supernatant was analyzed. The residue was washed twice with 8 mL ultrapure water.
The bound to Fe/Mn oxides of low crystallinity (Hg-OX)	0.4mol/LNH_2_OH·HCl (soluble in 20%HAc(*v*/*v*))	After treatment in step 2, add 40 mL extract, 96 °C water bath 6 h, centrifugal 20 min, take supernatant analysis. The residue was washed twice with 8 mL ultrapure water.
The bound to organic matter (Hg-OM)	H_2_O_2_ (HNO_3_ regulates pH = 2.0)	Residue after step 3 treatment, add 16 mL extract, 80 °C water bath 2 h, add 6 mL extract, 80 °C water bath 3h, centrifugal 20 min, take supernatant analysis. The residue was washed twice with 8 mL ultrapure water.
The residual (Hg-RES)	Aqua regia (HNO_3_:HCl = 1:3)	The residue was treated by step 4, adding 20 mL extractant, 95 °C water bath10min, adding 1.0 mL BrCl and 95 °C water bath 30 min again.

**Table 2:** Properties and components in soils of different profiles in the manganese mining and smelting areas.

Sampling Points		pH	SOM	CEC	Mn	Fe	S	Se
			(g kg^–^^1^)	(cmol^+^ kg^–^^1^)	(mg kg^–^^1^)	(mg kg^–^^1^)	(mg kg^–^^1^)	(mg kg^–^^1^)
Mining area (0–10 cm)	Maximum value	7.57	64.85	17.35	7535.80	42,335.40	709.43	2.35
	Minimum value	4.53	11.72	5.40	121.40	14,357.10	100.72	0.22
	Average value	5.97	29.69	10.50	1613.69	27,458.69	304.83	0.85
	Standard deviation	0.81	11.57	3.51	1879.65	6088.50	126.72	0.50
Smelting area (0–10 cm)	Maximum value	7.44	47.20	25.56	10316.60	53,781.70	933.24	1.37
	Minimum value	4.61	10.18	7.21	175.00	15,082.00	106.89	0.34
	Average value	6.35	28.84	12.13	1609.11	28,572.99	289.41	0.80
	Standard deviation	0.63	9.93	3.23	2415.33	9229.10	149.64	0.23
Mining area (10–20 cm)	Maximum value	7.93	43.79	16.92	8009.39	42,148.71	786.91	2.57
	Minimum value	4.33	13.94	3.60	85.16	14,161.04	111.64	0.26
	Average value	5.96	25.18	10.04	1682.66	27,877.52	296.15	0.82
	Standard deviation	0.89	6.81	3.21	1962.86	6526.59	141.38	0.43
Smelting area (10–20 cm)	Maximum value	7.67	55.94	24.30	10883.99	55,503.22	781.91	1.95
	Minimum value	4.54	10.48	6.54	193.28	14,716.25	88.33	0.46
	Average value	6.32	27.34	11.54	1508.11	28,694.20	275.10	0.80
	Standard deviation	0.72	9.70	3.26	2241.01	9486.01	134.25	0.26
Mining area (20–30 cm)	Maximum value	7.88	37.60	15.96	9625.27	46,274.67	432.42	3.02
	Minimum value	4.16	4.53	4.11	135.33	14,075.81	20.08	0.24
	Average value	5.93	20.95	9.76	1591.87	28,911.75	257.38	0.81
	Standard deviation	0.96	7.65	3.04	2007.18	6698.66	79.83	0.54
Smelting area (20–30 cm)	Maximum value	7.83	55.39	15.82	9259.29	63,838.91	497.77	1.98
	Minimum value	4.15	6.10	6.32	239.19	15,324.93	31.52	0.27
	Average value	6.56	22.14	11.08	1197.41	29,021.45	242.43	0.74
	Standard deviation	0.65	9.62	2.34	1572.37	9925.21	105.46	0.34
Mining area (30–40 cm)	Maximum value	7.81	35.47	17.27	10858.12	49,717.83	858.71	1.91
	Minimum value	4.18	5.53	3.37	106.05	15,132.86	31.26	0.17
	Average value	6.17	16.49	9.59	2019.54	30,971.28	228.46	0.64
	Standard deviation	0.85	7.71	3.22	2620.25	7377.17	135.62	0.35
Smelting area (30–40 cm)	Maximum value	7.89	55.80	29.89	7702.29	71,869.28	353.79	1.26
	Minimum value	4.24	2.70	6.52	210.48	12,082.49	14.04	0.33
	Average value	6.72	17.33	10.89	1115.55	31,531.45	189.73	0.61
	Standard deviation	0.64	10.36	3.46	1231.52	12,662.92	96.66	0.23

## Data Availability

Not applicable.

## Supplementary Materials

The following supporting information can be downloaded at: https://www.mdpi.com/article/10.3390/ijerph191610288/s1, Figure S1: Dendrogram of mining and smelting areas; Table S1: Comparison of Hg and MeHg concentrations in farmland soils.
