# Supplementary material for: Distribution of Mercury and Methylmercury in Farmland Soils Affected by Manganese Mining and Smelting Activities
Source: Int J Environ Res Public Health. 2022 Aug 18;19(16):10288. doi: 10.3390/ijerph191610288 (PMC9408302; doi:10.3390/ijerph191610288)
Supplement: Supplementary file 1 [file ijerph-19-10288-s001.zip › ijerph-1842307-supplementary.pdf]

Distribution of mercury and methylmercury in farmland soils affected by manganese mining and smelting activities

Yongjiang Zhang [1,2], Xian Zhou [3], Weibin Ma [2], Deliang Yin [3], Yongmin Wang [2], Cheng Zhang [2] and Dingyong Wang [2,*]

1 Department of Environment and Quality Test, Chongqing Chemical Industry Vocational College, Chongqing, 401220, China

2 College of Resources and Environment, Southwest University, Chongqing 400715, China

3 Key Laboratory of Karst Georesources and Environment, Ministry of Education, Guizhou University, Guiyang 550025, China

*** Corresponding: dywang@swu.edu.cn**

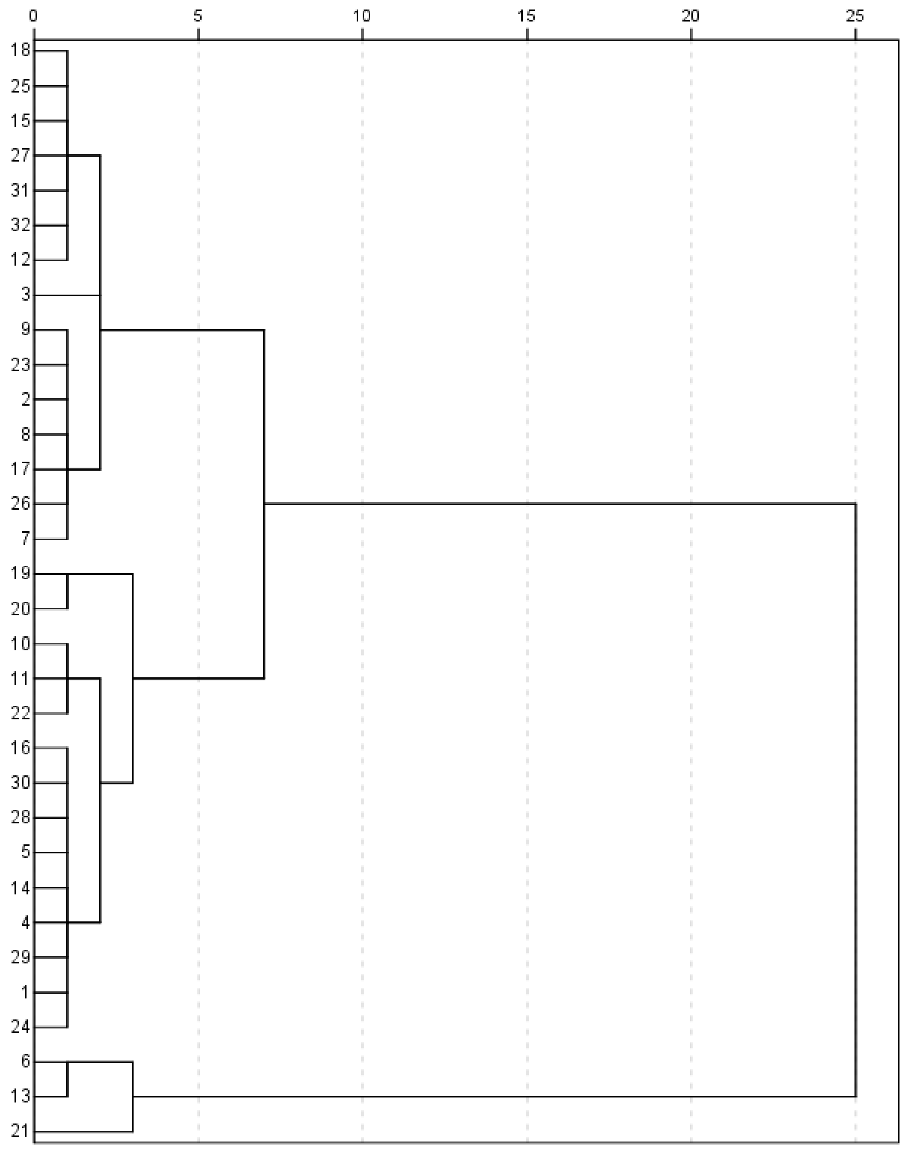

Figure S1. Dendrogram of mining and smelting areas

Note: Numbers 1-16 belong to the mining area, and numbers 17-32 belong to the smelting area

Table S1. Comparison of Hg and MeHg concentrations in farmland soils

Regions	Hg (mg kg^{-1})	MeHg (μg kg^{-1})	References
Manganese mining aera	0.54 ± 0.45	0.36 ± 0.39	This study
Manganese smeling aera	0.63 ± 0.55	0.88 ± 1.00	This study
Chongqing, China	0.069	/	Bao et al., 2020
Xiushan Hg mining area, China	9.8 ± 17.5	1.36	Xu et al., 2018
Wanshan Hg mining area,China	31	2.8	Yin et al., 2018
Chongqing, China	/	0.41 ± 0.19	Sun et al., 2019

[1] Bao L.; Deng H.; Jia Z.; Li Y.; Dong J.; Yan M.; Zhang F. Ecological and health risk assessment of heavy metals in farmland soil of northwest Xiushan, Chongqing. In Chinese.

Geol. China 2020, 47(06):1625-1636.

[2] Xu X.; Lin Y.; Meng B.; Feng X.; Xu Z.; Jiang Y.; Zhong W.; Hu Y.; Qiu G. The impact of an abandoned mercury mine on the environment in the Xiushan region, Chongqing, southwestern China. Appl. Geochem. 2018, 88:267-275.

[3] Yin D.L.; He T.R.; Yin R.S.; Zeng L.X. Effects of soil properties on production and bioaccumulation of methylmercury in rice paddies at a mercury mining area,China. J. Environ. Sci. 2018, 68(6):194-205.

[4] Sun T.; Ma M.; Du H.; Wang X.; Zhang Y.; Wang Y.; Wang D. Effect of different rotation systems on mercury methylation in paddy fields. Ecotoxicol Environ. Saf. 2019, 182:109403.
